# Celebrating 360 years of *Philosophical Transactions*

**DOI:** 10.1098/rstb.2024.0492

**Published:** 2025-01-09

**Authors:** Richard Dixon, Helen Eaton

**Affiliations:** ^1^ Department of Biological Sciences, BioDiscovery Institute, University of North Texas, Denton, TX 76203, USA; ^2^ Senior Commissioning Editor, Philosophical Transactions B, The Royal Society, London, UK

The 6th March 2025 marks the 360th anniversary of the first issue of *Philosophical Transactions*, and this Editorial launches a year of celebrations. Please look out for activities happening throughout the year to celebrate the long and distinguished history of the Royal Society’s journals.

The longevity of the journal is remarkable and largely explained by the important and special place it occupies in the history of science. As Thomas Henry Huxley famously stated in 1866: *‘if all the books in the world except the Philosophical Transactions were destroyed, it is safe to say that the foundations of physical science would remain unshaken, and that the vast intellectual progress of the last two centuries would be largely, although incompletely, recorded*’ [1, p. 23]. The journals have survived many transformations since Henry Oldenburg, Secretary of the Royal Society, decided to publish a periodical to promote ‘natural philosophy’: the introduction of peer review, the proliferation of scientific literature and the digital revolution, to name but a few.^1^

Oldenburg’s first Editorial, published in March 1665 [2], stated why he felt it important to launch the publication, and in many ways it still reflects the objectives of publishing today: communicating and celebrating scientific research, sharing ‘solid and useful knowledge’ and providing a basis for others to build on ideas, so that people can ‘*contribute what they can to the Grand design of improving Natural knowledge … [for] the Universal Good of Mankind*’.

*Whereas there is nothing more necessary for promoting the improvement of Philosophical Matters, than the communicating to such, as apply their Studies and Endeavours that way, such things as are discovered or put in practice by others; it is therefore thought fit to employ the* *Press* *as the most proper way to gratifie those, whose engagement in such Studies, and delight in the advancement of Learning and profitable Discoveries, doth entitle them to the knowledge of what this Kingdom, or other parts of the World, do, from time to time, afford, as well of the progress of the Studies, Labours, and attempts of the Curious and learned in things of this kind, as of their complete Discoveries and performances: To the end, that such Productions being clearly and truly communicated, desires after solid and useful knowledge may be further entertained, ingenious Endeavours and Undertakings cherished, and those, addicted to and conversant in such matters, may be invited and encouraged to search, try, and find out new things, impart their knowledge to one another, and contribute what they can to the Grand design of improving Natural knowledge, and perfecting all Philosophical Arts, and Sciences. All for the Glory of God, the Honour and Advantage of these Kingdoms, and the Universal Good of Mankind.*

Henry Oldenburg [2, pp. 1–2]

The history of the journal can be explored in more depth in the landmark ‘Science in the Making’ project: https://makingscience.royalsociety.org. On this website, you can discover some of the incredible archives that lie behind the published articles: peer reviews, correspondence, photographs, illustrations and early drafts. The website is a unique chance to understand how the scientific world has operated over time and how things have changed.

As I (RD) talked about in an Editorial last year [3], integrity in publishing is at the core of how the Royal Society journals operate (and from the start of 2025, we will have a dedicated integrity advisor on the journal’s Editorial Board). During 2025, as part of the anniversary celebrations, we will be reflecting on the state of the publishing industry and the challenges that we face. Look out for blogs and webinars over the coming months.

Peer review continues to be a cornerstone of scientific publishing. The Royal Society was the first scientific organization to implement a policy whereby all papers would be reviewed, as announced in the Presidential address of 1832 (given by then President Prince Augustus Frederick, Duke of Sussex; [4]). The problem of reviewer fatigue was recognized even in these early days—as the announcement states *‘When the number of papers which come before the Society in the course of a year is considered, as well as the great diversity and occasional difficulty of the subjects which they embrace, it will be at once seen how greatly the labours and responsibility of the Members of the Council must necessarily be increased by the rigorous adoption of such a system*’. The President had a very high level of confidence in the Fellows of the Royal Society to undertake their role as reviewers: *‘The decisions of men who are elevated by their character and reputation above the influence of personal feelings of rivalry or petty jealousy, possess an authority sufficient to establish at once the full importance of a discovery, to fix its relations to the existing mass of knowledge, and to define its probable effect upon the future progress of science*’.

In the spirit of increasing transparency, we hope to start publishing reviewers’ reports and other materials alongside published articles in the coming months in *Philosophical Transactions B*. Formats of peer review such as open peer review and collaborative review are usually seen as very modern, experimental forms of peer review, but in fact, the Royal Society was experimenting with this in its early days of reviewing—see for example the 1832 published report written by William Whewell and John William Lubbock, of a paper by Astronomer Royal George Biddell Airy [5]. The value and interest of the reviewer reports were recognized—as the President wrote in his 1832 address ‘*the Reports which are thus produced prove often more valuable than the original communications upon which they are founded, and the collections of them, as is well known, form a most important part of the stock of modern science*’. The collaborative nature of the trial proved a struggle. The additional time and effort to prepare these reports collaboratively, and in a fashion that was fit for publication, deterred the editorial committee from continuing this open practice after a couple of years.

It is fitting that this first issue of 2025 is dedicated to Professor Georgina Mace. Georgina, who very sadly died in 2020, was the Editor in Chief of *Philosophical Transactions B* in 2010 when the Royal Society celebrated its 350th anniversary. It is worth looking back at the special issue that Georgina put together to mark this anniversary: ‘Personal perspectives in the life sciences for the Royal Society’s 350th anniversary’ [6].^2^ This issue asked leading researchers across the biological sciences to reflect on their areas of research. The topics cover the broad scope of biological science covered in the journal, and readers may find it interesting to consider how things have progressed in the last 15 years.

Georgina Mace was the first female Editor in Chief of a Royal Society journal when she took on the role in 2008. The dedication of the first issue of 2025 to celebrating her work coincides not only with this journal’s anniversary year but also marks 80 years since the first women, crystallographer Kathleen Lonsdale and biochemist Marjory Stephenson, were admitted to the Royal Society Fellowship. The Royal Society will be celebrating this event with activities during 2025. You can read more about some of the fascinating stories of our female Fellows in a special collection of memoirs: https://royalsocietypublishing.org/topic/special-collections/celebrating-women-in-science.

*Philosophical Transactions B* has worked hard to increase the number of women involved with the journal. Today, over 50% of our Editorial Board are women, and in 2024, 39% of our Guest Editors were women and 44% of our submitting authors. Gender, age and geographical diversity are critical for the continued success of this journal, and we have been working hard to increase representation from groups that have not previously published with us. We are very pleased that in 2025 we will have Guest Editors located in every continent (apart from Antarctica), and we hope that this will also be reflected in the authorship of the papers that we publish. We welcome submissions of proposals for theme issues from researchers in any area of the world and at any career stage, so please do keep us in mind if you have an idea. Find out more at https://royalsocietypublishing.org/rstb/submit-proposal.

The technological developments of the past 360 years have given us a world that appears a smaller place but where issues are more complex. Science affects all of us; improving natural knowledge is indeed a ‘Grand design’ and the journal is dedicated to pursuing Oldenburg’s vision of providing a forum for all those who are ‘addicted to and conversant in’ biological discoveries, that will now shape the twenty-first century.

## Data Availability

This article has no additional data.

## Footnotes

^1^
Find out more about the history of the journal at https://royalsociety.org/journals/publishing-activities/publishing350/history-philosophical-transactions.

^2^
Theme Issue 'Personal perspectives in the life sciences for the Royal Society’s 350th anniversary' compiled and edited by Georgina Mace, https://royalsocietypublishing.org/toc/rstb/2010/365/1537.
